# Review and Suggestion of Failure Theories in Voids Scenario for VARTM Processed Composite Materials

**DOI:** 10.3390/polym13060969

**Published:** 2021-03-22

**Authors:** Vivek Kumar Dhimole, Pruthvi Serrao, Chongdu Cho

**Affiliations:** Department of Mechanical Engineering, Inha University, 100 Inha-ro, Michuhol-gu, Incheon 22212, Korea; VIVEK.DHIMOLE@INHA.EDU (V.K.D.); serrao@inha.edu (P.S.)

**Keywords:** failure criterion, vacuum-assisted resin transfer molding (VARTM), void defects, composite materials, material processing

## Abstract

Fiber-reinforced composite structures are used in different applications due to their excellent strength to weight ratio. Due to cost and tool handling issues in conventional manufacturing processes, like resin transfer molding (RTM) and autoclave, vacuum-assisted resin transfer molding (VARTM) is the best choice among industries. VARTM is highly productive and cheap. However, the VARTM process produces complex, lightweight, and bulky structures, suitable for mass and cost-effective production, but the presence of voids and fiber misalignment in the final processed composite influences its strength. Voids are the primary defects, and they cannot be eliminated completely, so a design without considering void defects will entail unreliability. Many conventional failure theories were used for composite design but did not consider the effect of voids defects, thus creating misleading failure characteristics. Due to voids, stress and strain uncertainty affects failure mechanisms, such as microcrack, delamination, and fracture. That’s why a proper selection and understanding of failure theories is necessary. This review discusses previous conventional failure theories followed by work considering the void’s effect. Based on the review, a few prominent theories were suggested to estimate composite strength in the void scenario because they consider the effect of the voids through crack density, crack, or void modeling. These suggested theories were based on damage mechanics (discrete damage mechanics), fracture mechanics (virtual crack closure technique), and micromechanics (representative volume element). The suggested theories are well-established in finite element modeling (FEM), representing an effective time and money-saving tool in design strategy, with better early estimation to enhance current design practices’ effectiveness for composites. This paper gives an insight into choosing the failure theories for composites in the presence of voids, which are present in higher percentages in mass production and less-costly processes (VARTM).

## 1. Introduction

Composite materials have significant properties like their high strength to weight ratio [1,2,3]. They are used in many industries for different complex structures, such as aircraft, automobile, marine, and sports. Polymer matrix composites (CFRP and GFRP) are widely used composite materials [4,5,6]. Different manufacturing processes are used to fabricate composite parts like hand layup, autoclave, out-of-autoclave (OoA), and additive manufacturing (AM) [7,8,9]. The autoclave is a highly labor-dependent, time-consuming, and costly process not suitable for mass production. Additive manufacturing is also a well-known fabrication technique based on adding material. Now, this process is also improved at a significant level for better quality. However, for large and bulky products, this will be costly, time-consuming, and can affect shape quality. Postprocessing is another issue in this process [10,11]. Out-of-autoclave processes are less costly and based on oven/mold [12,13]. Liquid composite molding (LCM) is an appropriate OoA technique that can produce complex shapes with high quality and quantity. Resin transfer molding (RTM) specifically is mostly accepted among industries, having numerous advantages to manufacture complex aero-engines and automotive parts [14]. The matched metal tooling process in the RTM is costly, and for manufacturing large and bulky structures such as boat hulls, tool design is complicated. Vacuum-assisted resin transfer molding (VARTM) was developed to modify the traditional RTM process to diminish the cost and design difficulties linked with bulky metal tools. Due to the cheap and mass-productive advantage of the VARTM process, it is developing day by day.

Initially, the VARTM cycle’s advancement was introduced by Williams, Summerscales, and Gove in their noteworthy review of resin injection under flexible tooling (RIFT) [15]. In 1950, The VARTM process was presented, and furthermore, vacuum infusion process (VIP) or resin infusion under flexible tooling (RIFT) were investigated. Later, different groups worked for a variant of VARTM like Lotus Car Ltd. Then, in 1980 and 1985, Gotch extended and replaced one half of the mold with a silicone vacuum rubber bag, incorporating vacuum pressure to draw the resin from a resin supply into the dry preform [16]. In 1990, Seemann patented the Seemann composite resin infusion molding process (SCRIMP) as a variant of the VARTM process, which is widely used for manufacturing large composite structures [17]. Fast remotely actuated channeling (FASTRAC) is another developed variant of the VARTM process to reduce waste [18]. Some other studies were also recommended to spread external compaction pressure. such as the inflatable bladder, permanent magnets, or pressurized air to reduce process-induced voids and increase fiber volume fraction [19,20,21]. Despite that, due to the restriction of estimating clamping powers, these recommended variations of VARTM are limited to small and medium-size composite parts. Other variants, such as double bag vacuum infusion (DBVI), vacuum-assisted process (VAP), and controlled atmospheric pressure resin infusion (CAPRI), were also developed and patented to enhance repeatability of the process and to minimize void contents and the intrinsic thickness gradient [22,23,24]. 

However, because of the mass and cost-effective production environment, the VARTM manufactured products contain voids and micro-voids as ordinary deformities which influence the composites’ applications [25,26,27]. The production of voids and flaw-free composites will be costly and not feasible, so it is necessary to consider the effects of voids defects and their progression, even in the design cycle’s beginning phases. Different experimental or analytical studies have proven that these defects are responsible for initiating and evolving damage actions and then certainly affect the critical state of final failure [28,29].

It is an important topic to determine failure state and components’ effectiveness due to growth in the advanced application of composite structural materials in current years. When the final processed composites present flaws, then it is necessary to incorporate manufacturing defects in failure theories. There are many failure criteria for composites which have been developed without consideration of voids. These are summarized in Section 3. Researchers have started to work and estimate the strength of composites with manufacturing defects. Many studies have shown that voids diminish the transverse tensile and compressive strength of laminate at the application level and decrease the trustworthiness of manufactured composites. Composite defects should be taken into consideration because they influence many functionalities of composite products. Air voids are the most common influenceable flaws, significantly affecting the application and eligibility of composite structures [30]. Experimental studies showed that voids and microcracks are the common known defects in textile composite materials caused by the manufacturing process [31].

Void-free manufacturing is impossible, but to work safely at the application level, reliable design is necessary [25,27,32]. That’s why today’s performances under defects are studied, and different approaches are coming into the picture to make it feasible [33]. The suitable failure theory could be incorporated into the performance evaluation process when the defect information is gained from previous material characterization and manufacturing processes.

**Figure 1:**
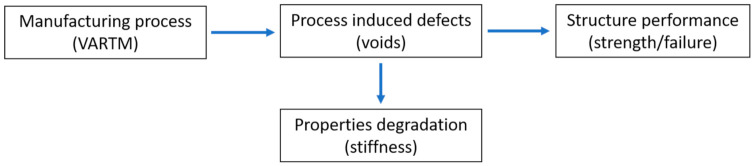
Process and effect of defects (voids).

Figure 1 shows the manufacturing process and its defect and effects of defect on manufactured components. This review presents the VARTM process (polymer matrix composites) as cost-effective, failure theories with and without considering voids, the need to consider voids, and suggested failure theories to consider defects (voids). Some suitable failure theories have been discussed comprehensively, which are capable for composites in voids scenario and safe the design from misleading. Every suggested criterion has its advantage for a designed application. A strain-based criterion was suggested for a primary or initial check of failure. Discrete damage mechanics is suitable as it considers voids as a crack or void density and its evolution function. Fracture mechanics (virtual crack closure technique (VCCT)) was suggested for delamination, which is the case of void growth, and a micro-mechanics study was discussed for the estimation of failure at the micro level due to voids and their effect on macro-failure. These theories were suggested to consider the effect of voids in composite failure design, and these are also well established in the finite element method (FEM) to save time and costs for predesign. Based on this review for failure theories, one can obtain the importance of manufacturing conditions in the design strategy, and in the future, further enhanced and accurate criteria can be established considering manufacturing effects like voids among others.

## 2. VARTM Process

The vacuum-assisted resin transfer molding (VARTM) process has been developed to use effectively during the past two decades. This closed-mold technique can produce high performance and various types of composites, especially fiber reinforced polymer structures, at low cost [34]. The method involves primarily putting the fibers or cloth fabrics in a preform in the desired configuration. Often, these fabrics are held together by a binder and pre-pressed to the mold shape. A top (second) matching mold tool is clamped over the first and vacuum-sealed used as a deformable vacuum bag. Then, the pressurized resin is injected into the cavity by the aid of vacuum [35].

**Figure 2:**
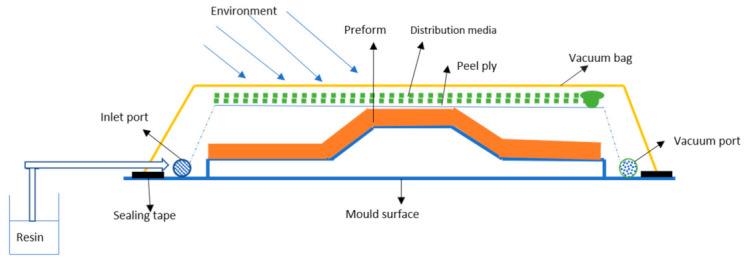
VARTM process setup layout.

Afterward, the laminate is healed, and both injection and cure can occur at either ambient or elevated temperature. In this process, it is possible to use any fibers, and the stitched materials work well since the gaps allow for the rapid transport of resin [36]. This is a flawless process using low-cost composite materials without prepregs and autoclaves compared with the conventional composite fabrication process used in the aeronautical field. The resin cup is typically open to the atmosphere, creating a pressure differential between the inlet and exit, causing the resin to be drawn into the layup [37]. Figure 2 shows a typical VARTM process setup.

Broadly, the VARTM technique can be separated into three processing steps, including material and tool preparation, viz. pre-infusion, infusion, and post-infusion [38]. Each process steps influence the final material quality, mostly the fiber volume fraction and void content distribution. Table 1 shows three-step process completion with the reason for voids inclusion in each process step.

There are different names to describe this process, viz. vacuum-assisted resin infusion molding (VARIM), vacuum-assisted resin infusion (VARI), and vacuum bag resin transfer molding (VBRTM). Further, many other developments have been in the VARTM process, such as Seeman composites resin infusion molding process (SCRIMP), vacuum-assisted process (VAP), and controlled atmospheric pressure resin infusion (CAPRI).

### 2.1. Advantage

**Figure 3:**
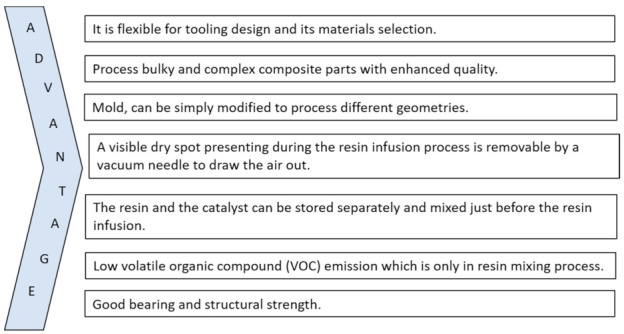
Advantages of VARTM.

The VARTM procedure was formerly produced to fabricate superior and huge composite parts, such as infrastructure and transport structures [38,39]. This process is cheap and established for mass production, and the basic principle is to develop the pressure difference between the vacuum and environment pressure to get the desired necessities. The points of advantages in the VARTM process are summarized below in Figure 3.

The main benefit of VARTM is that it lies in the low injection pressures (approx. 1 atm). At the time of manufacturing, less movement is required for reinforcement, which has been achieved by this little pressure to get better quality products [40]. Due to the advantage and improved understanding of process physics, VARTM is employed in various railroads, naval, aerospace, and automobile parts with many variations of VARTM, and more complex composite parts with higher quality, strength, and lower cast can be produced [34,38]. Although there are advantages of VARTM process and it is used widely, for accurate design, its primary defects’ (voids) effect should be known and considered alongside the formation of voids, as summarized below.

### 2.2. Defects in VARTM Processed Composites 

**Figure 4:**
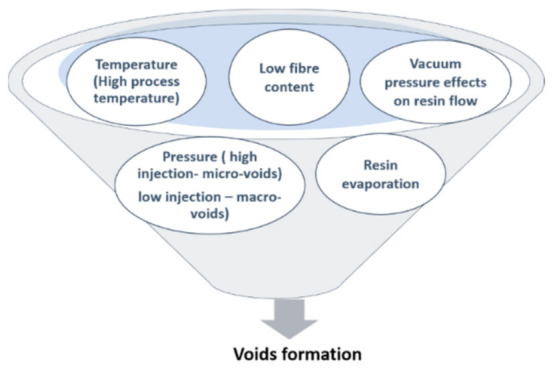
Voids formation factors.

**Figure 5:**
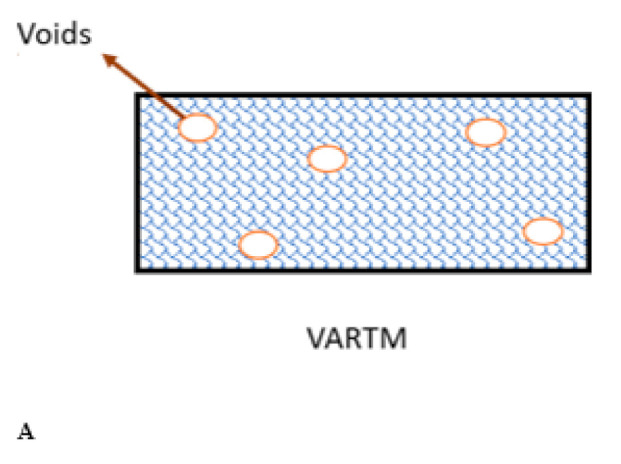

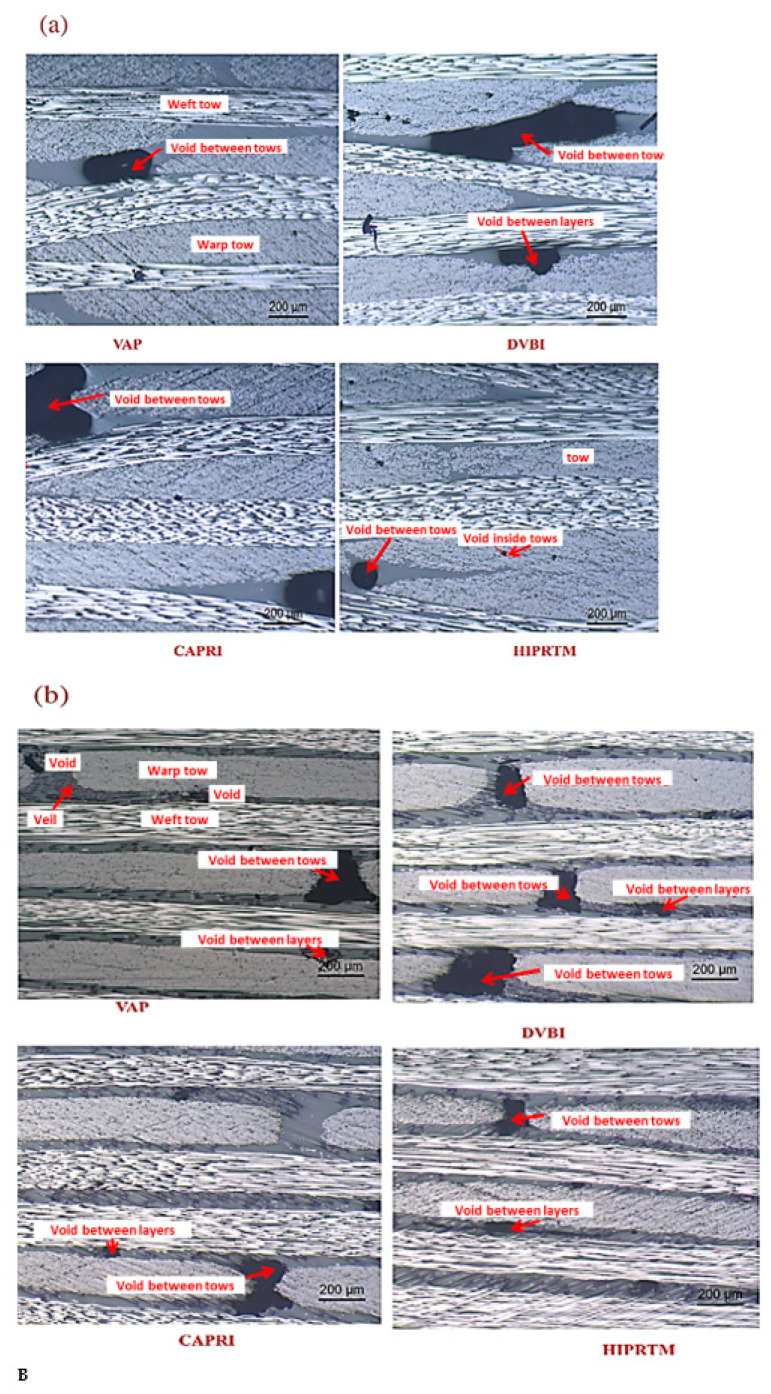
(**A**) Voids demonstration (Schematic), (**B**) (**a**) Voids in different VARTM process for woven reinforcement, (**b**) voids in different VARTM for non-crimp reinforcement adapted from [14], with permission from Elsevier, 2021.

Over the VARTM process’s advantages, there are different types of defects, like fiber misalignment and voids, whereby defect-free manufacturing is impossible. In mass production, voids formation is the primary defect. Many factors like resin-flow pressure variation or temperature changes initiate voids formation in this process [41,42]. Figure 4 shows void formation factors in the VARTM manufacturing process. In the VARTM process, void content is a significant issue in mass production and a cost-effective environment [26]. It reduces strength and the modulus which creates an early failure of composites in a loading environment [25,27]. This is why taking care of voids in failure theories remains very important to safe instead of misleading design. Figure 5A shows the voids scenario’s schematic in the VARTM processes to demonstrate a high void content in the process. Figure 5B illustrates actual images of voids in different VARTM processes for woven (a) and non-crimp (b) reinforcement from experiments [14]. This figure indicates that the voids’ conditions are higher in the mass production process (VARTM). This is why it is necessary to consider the manufacturing details (voids effect) in failure design conditions.

## 3. Failure Theories

Linear models, including maximum normal stress, maximum strain, and maximum shear stress criterion, were the earliest failure models for composites and brittle/ductile materials. Failure theories for the composite are different from ductile and brittle materials. Norris presented a simple anisotropic failure model for plywood [43]. Hill extended Mises criteria for orthotropic, but the main drawback is the lack of Bauschinger effect [44]. Tsai extended the same term. Tsai and Wu showed the quadratic function to take stress into account, but for calculating parameters, complex and expensive tests are required. Moreover, a single equation cannot define all failure modes, so Hashin developed the criteria for different failure modes [45,46]. Later, Pang et al. tried to reduce experimental work based on micromechanical and constituent properties consideration [47]. Previously presented quadric failure criteria were modified by Yeh et al. to reduce the complex bi-axial experimental work to determine the interaction terms’ coefficients. Bearing this in mind, Puck and co-workers developed a new failure theory for fiber-reinforced composites based on Mohr’s hypothesis and its modification by Paul [48,49,50]. In 1996–2004, the world-wide failure series was started with well-known, established theoreticians and designers of failure theories. Nineteen theories were summed up by this exercise and concluded only a few theories met the results with test cases, and for triaxial loading world-wide failure two was organized with 12 test cases [51,52]. In 2014, world-wide, failure three was organized with five different loadings and 13 cases. World-wide failure summarized many general theories, some of which offered better predictions with test cases [53]. In the 1990s, a review of the fibrous composite material was presented with different polynomial, interactive, Hart Smith, and Kring failure models [54]. Daniel presented a short description of failure theories viz. interlaminar and ultimate laminate failure with focusing textile composites. Recently review on interactive and non-interactive with progressive failure was published [55]. Table 2 shows the different failure theories and their working approach.

Till now, the above-described failure prediction theories have not considered any manufacturing defects such as voids. As discussed, voids are well-known defects in VARTM processed composites, so the understanding of failure with the void’s effects is necessary to access good design. This was started earlier to consider manufacturing defects in the failure mechanism. The next section covers a precise survey on failure theories with defects (voids).

### 3.1. Failure Theories with Considering Manufacturing Defects (Voids)

The above-presented theories did not take the voids defects into account, so this section covers the works that consider the voids’ effect. Chamis introduced a two-level failure theory with voids for unidirectional filamentary composites, matrix strain magnification, and introduced a rule of mixture for the first level and modified distortion energy for the second level [81]. These theories incorporated void contents and checked the strength of different composites considering void fraction. This research proposed that fabrication process effects such as voids are necessary to account for strength estimation and obtained a significant transverse and shear strength drop with 5% void for Thronel-50 and boron composites. Also, it provided the theoretical formulation to consider the effect of voids. Varna et al. studied the effect of voids on unidirectional composites under transverse load and obtained the effects of high and low void contents on a transverse crack and showed the effect of the voids’ shape on failure [82]. Further, it was proposed that transverse strain rates were affected by the voids configuration for transverse failure and found that crack and stress concentration irregularity happened due to voids which change the failure states. The void’s effect on transverse strain rate failure conditions was also obtained, i.e., the high void content laminate has higher transverse strain to failure (approx. 2%) than lower void content transverse strain to failure (approx. 0.3%). Jeong studied the effect of voids on strength and attenuation slope for unidirectional and woven graphite fiber-reinforced composite and concluded that unidirectional composites’ strength is more sensitive than woven composites in the presence of higher voids content [83]. It was further found that there is a relation between attenuation slope with void content and interlaminar shear strength for laminates and stated that strength was reduced with increasing attenuation due to voids. Colavito et al. studied void effects on strength and suggested prydonomic theory to consider voids and verify that by indentation tests [84]. The presented theory is well qualified for damage estimation in the presence of voids and it was obtained that failure was mostly matrix dominated. At the front and back areas of composites, laminates were analyzed and damages were more serious at the laminates’ back surface. Ricotta et al. presented a process to consider void effects on the mode I strain energy release rate model with FEM [85]. They studied voids shape, size, and location and showed that with the increment in size SERR increase, and elliptical voids are more severe than circular voids also found the effect of small voids is higher at the crack tip than large voids far from the crack tip. Choudhary et al., studied the void’s effects for crack initiation and growth with energy-based criteria for a failure mechanism under transverse loading to fibers [86]. They showed the void’s effect on micro to macro-level failure with the initiation of growth, crazing model, and debonding model. Found the void’s size and fiber spacing effect, microlevel features, and voids reduce the strength while promoting crazing phenomena, making failure at large strain. Zhuang studied in his thesis that void percentage affects delamination growth under compression load [87]. He found the presence of mixed-mode delamination for under compression loading, which is the case of out of plane buckling and obtained that the effect of voids is more prominent on mode II SERR than mode I SERR, dependent on the void’s size and location. Talreja presented delamination and transverse crack in a cost-effective environment, with strain energy release rate crack density in an element and Weibull function for stress distribution in plies [88]. They found crack density to be higher in large air-entrapped (voids) than low voids, and according to this finding, suggested that mode and shape of failure are dependent on defect (voids) condition, so the void’s shape, size, and location should be considered for failure estimation. Shigang et al. showed a strain-based damaged model to predict the progressive failure and fracture (controlled by energy and strain) of a composite with voids (by the Monte Carlo algorithm for random selection to reach void percentage and used micro-computed tomography data) [89]. After investigation with a woven C/C composite, it was found that voids in fiber yarn affect the tensile strength more than voids in the matrix or interface, so a continuity in fiber is necessary during manufacturing. Liu and Chen published a review for voids formation, its evaluation, and its effect on interlaminar shear, compressive, flexural, and fatigue strength [42]. They further showed the effect of different conditions on voids, such as temperature and pressure, and summarized that matrix-dominated properties affected more than fiber-dominated in the presence of voids. It was also suggested that shape size and location of voids also play an important role in strength. Suhot et al. used acoustic emission and X-ray tomography to assess the effect of voids on the flexural strength of a carbon epoxy composite [90]. They found that 2% increment in voids reduced 12.7% flexural strength and showed that void’s size has a major role in reducing strength and initiate delamination. Xu et al. modeled the FE model with random voids effects and presented the void in the FE model through the monte-carlo algorithm, and used fracture energy criteria to take them into account for strength prediction [31]. They proposed that voids have more effect on elastic modulus and tensile strength of matrix than fiber yarn in braided C/C composites. Liu et al. introduced an FE study with equivalent strain criteria and modeled voids by image analysis using the MATLAB image processing tool for damage and strength estimation [91]. They found that in carbon fiber/polymer laminates, strength and stiffness were degraded in the presence of voids, observing a different void percentage (8.9%, 0.6% and 3.8%) effect with higher void content (8.9%) initiating damage at a lower loading than the lower void percentage and the voids’ effect being different directional strength. Sudhir and Talreja discussed the micromechanics approach with brittle cavitation and induced matrix defects in RVE for a transverse crack through the FE modeling of voids [92]. They found the aspect of brittle cavitation in an epoxy matrix for the transverse crack in fiber-matrix debond, also obtaining that due to voids transverse cracks arise in fiber epoxy composites. Xu and Huang proposed a mesoscale FE model with voids and illustrated a tensile fracture with a concrete’s continuum damage model [93]. They obtained that voids affect pre-peak and post-peak conditions, and two types of failure modes (single and two macro-scale crack) were summarized. Jiang et al. presented three representative unit cell (RUC) FE models with voids and used stress, stassi, and stiffness degradation failure criteria with different loading and concluded matrix failure domination and showed transverse strength effects more than shear strength in the presence of voids [94]. They studied the effect of voids with 0.15%, 0.5%, to 5% on a carbon fiber reinforced composites’ bundle and proved that voids diminish the strength and stress concentration depending on loading directions. Dong proposed a simple regression model after fitting the finite element analysis (FEA) results and used that model to predict composite laminates’ strengths with considering voids [41]. They obtained that the voids effect matrix dominated transverse properties, with a % increment of voids accompanied by a 1.5% decrement of transverse modulus, but longitudinal modulus was effected less. Sun et al. studied kinking failure and the influence of voids on that, and obtained that voids are the cause of deflection and splitting of kink-band [95]. Through microscopic images, it was found that failure initiation is the cause of pre-notching and obtained that during the manufacturing process, kink band form because of voids, and these voids lead to kink band splitting and deflection in a different direction. Recently, Hyde et al. presented matrix and interfiber voids and showed the effect of these voids on failure under transverse, compression, tension, and shear loading, and discussed the effect of the size and location of voids on failure [96,97]. They found that voids propagate for damage at the microlevel. In shape, pentagonal interfiber voids affect more than square interfiber and circular matrix voids on strength, and void geometry affects the stress concentration. They also proposed that void orientation has less effect than fraction and shape.

**Figure 6:**
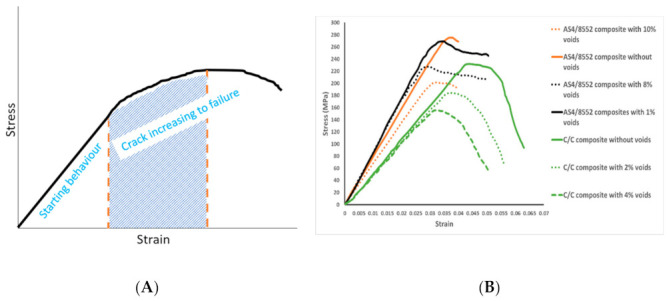
(**A**) Stress-strain response for understanding failure behavior, (**B**) Stress-strain response with and without voids.

In this section, the discussed survey took the effects of voids on failure mechanism. Although the above-discussed survey worked with different kinds of composites, it showed how the researcher took voids in failure theories. These researchers signify the reason to take manufacturing defects into account for a better pre-estimation of design. The different kinds of approaches were discussed. Some belong to experiments and others to analytical and numerical observations. When the load is applied on parts, if components cannot resist it, failure initiation starts, and when there are already voids, this failure initiation happens on less stress. Figure 6A is for the demonstration purpose and shows starting estimation of stress status under strain condition then post status when void/crack start to initiate and tends to failure. Figure 6B shows the stress–strain response for different composites in the presence of voids (dashed line) and without voids (solid lines), which also indicates the effect of voids on strength. So, voids play an important role in failure design [89,96,97]. From this understanding and getting the idea from surveyed failure theories, some suitable approaches were suggested for composites. These criteria are helpful to the design strategy of a mass-productive and cost-saving environment. The criteria were suggested because these were also well established in FEM, and covered the crack and voids details. Firstly, a strain-based criterion was suggested for the primary checking of component condition, then damage, fracture, and micromechanics based assessments were suggested to get the failure behavior in the presence of voids. These suggested criteria are discussed in the next section.

### 3.2. Proposed Failure Criteria 

#### 3.2.1. Strain-Based Theory

A strain-based criterion was proposed for the primary level check. The drawback in stress-based criteria is that scale effects associated with crack length with similar stress fields cannot be modeled correctly [98]. Also, due to voids, the elastic modulus decreases after loading in laminates. That’s why stress criteria cannot predict exact behavior accurately. Also, strain-based criteria are neither scalar nor vector, so direction dependency is not an issue that is beneficial than stress-based (vector) criteria. The proposed criterion for the primary check is discussed below.

##### Truncated Maximum Strain Criteria

In the aircraft industry, maximum strain criteria are well-established. On that basis, Hart-smith made advancement in maximum strain criteria by truncating its tension-compression coordinate for shear and compared with different direction laminate. These criteria made better failure prediction and also followed by aerospace industries [99]. Truncated maximum strain criterion could be used for starting estimation of failure at primary level, as shown in Figure 6A. This criterion is based on shear cutoff at the shear coordinate of maximum strain criteria, which means the truncated-maximum-strain criterion limits the strains in the tension-compression quadrants to account for the shear failure of the fibers, as shown in (Equation (3)). This is the advance step from maximum strain failure criteria [100].

(1) $$\varepsilon_{1c}<\varepsilon_{1}<\varepsilon_{1t}$$

(2) $$\gamma =\left|\varepsilon_{1}\right.-\left.\varepsilon_{2}\right|$$

(3) $$\left|\varepsilon_{1}\right.-\left.\varepsilon_{2}\right|=(1+\nu_{12})\max(\varepsilon_{1t},\varepsilon_{1c})$$

where $\varepsilon_{1c}$, and $\varepsilon_{1t}$ are the maximum compressive and tensile strain in the fiber direction (here, 1 is in the fiber direction), $\varepsilon_{1}$ is the logitudinal strain, γ is the shear strain, and $\nu_{12}$ is the in-plane Poisson ratio. Equations (1) and (2) define failure criteria in longitudinal and transverse directions, respectively. The truncated-maximum-strain criterion does not use *Ɛ*_2_ to check matrix cracking, but it works to check fiber failure in the complementary lamina, which can be built and model into the laminate precisely to constrain the transverse deformation and strain (*Ɛ*_2_). This criterion is well-established and straightforward to apply for researchers and industrialists within the FEM domain. More details about this criterion and its effectiveness can be found in the literature [99]. This was suggested for primary check because it is an enhancement of the well-known maximum strain criteria.

#### 3.2.2. Discrete Damage Mechanics (DDM)

Continuum damage mechanics studied by Kachanov for stress conditions in damaged metals under creep scenario; then this approach got researcher’s attention and developed for brittle, ductile, and composite materials. This approach represents the cracks indirectly by their effect on stiffness, using a damage variable, e.g., D_1_, D_2_, D_6_, etc., for orthotropic damage [101]. These damage variables cannot be measured directly, but indirectly through the reduction of stiffness. The hardening exponent or other constants for a kinetic equation control the damage growth and the parameters for kinematic equations are also difficult in CDM, while the finite element implementations of CDM are mesh dependent [102]. Although CDM is well established in industries and research, due to the above-discussed fact, DDM was suggested. It calculates the appearance of the void/crack condition, then the evolution of its density that changes the distribution of stresses in laminates because of the degradation of the laminate’s stiffness.

In DDM, the state variable represents a measurable quantity, i.e., the crack density lambda (λ). The analytical-numerical formulation solves the displacement field around the crack and in the whole laminate [103]. DDM calculates the energy release rate G from the displacement field and uses the Griffith–Irwing criterion to decide the crack density increasements. In comparison to CDM, the material constants are real and measurable (available by experiments or literature) for modes I and II of crack opening and sliding. Damage rate and stress–strain softening are calculated in terms of *G_c_* (critical fracture energy). Finite element implementation of DDM is mesh insensitive such that is most beneficial for computational study. The damage activation function is the function of strain and cracks density for the evolution of cracks or voids’ stage as shown in the below equations [104].

(4)  λ= 1/(2l)

(5) $$\begin{array}{l} g=(1-r)\sqrt{\frac{G_{I}(\lambda ,\varepsilon )}{G_{Ic}}}+r\frac{G_{I}(\lambda ,\varepsilon )}{G_{Ic}}+\frac{G_{II}(\lambda ,\varepsilon )}{G_{IIc}}-1\leq 0\\ r=\frac{G_{Ic}}{G_{IIc}} \end{array}$$

In Equation (4),  λ is the symbol of crack density, 2l is the inverse distance between two adjacent cracks. In Equation (5), *G_Ic_* and *G_IIc_* are the fracture toughness in mode I and mode II, respectively which are the material properties of given materials, *G_I_* and *G_II_* are energy release rates in mode I and mode II, respectively, and *r* is the constant term which is the ratio of *G_Ic_* and *G_IIc_*. *G_I_* and *G_II_* are calculated as the function of crack density (λ) and strain (ε). The crack density is defined by the inverse distance between two adjacent cracks, as shown in Equation (4). λ is the state variable and valuable to calculate the damage state in the cracked lamina. Equation (5) presents the crack evolution (g), which includes fracture toughness properties (*G_Ic_* and *G_IIc_*) and energy release rates (*G_I_* and *G_II_*) [105]. In DDM, a crack in lamina is represented by crack density, and first, it affects the individual lamina, then goes to laminate.

Equation (5) shows that when there is no crack (crack density  λ = 0), then the damage activation function will also be zero (*g* = 0), but when crack initiates, crack density and strain increase, then energy release rate (*G_I_* and *G_II_*) decrease, thus damage activation (*g*) will be less than zero.

DDM updates the state variable with the help of crack density and the strain, then calculates the resultants stress and tangent stiffness matrix of the lamina, so these are the functions of crack density. This criterion is validated extensively by experimental data from different sources for different kinds of materials and laminates. It can predict crack density and the reduction of modulus as a function of stress or strain and crack density accurately.

Kosteski et al. used a truss-like discrete element to model a crack; this was done by the duplicate node on the surface and eliminating the element near the crack material [106]. The above-reviewed facts concluded that this criterion is well established and accurate. More details of this criteria can be found in the literature [104,107].

#### 3.2.3. Fracture Mechanics (Virtual Crack Closure Technique (VCCT))

Griffith fracture concept in 1920, followed by Inglis, is essential in fracture mechanics [60]. It is formulated that when the existing crack grows and eventually becomes critical, a fracture will occur. Even though Griffith was able to formulate his ideas, his work was not noticed at that time due to the world war’s exigencies. Because of different scenarios such as plastic flow in ductile material, Griffith’s theory is not suitable for ductile materials. Irwin extended fracture theory for ductile material and gave the crack intensity factor parameter near the crack tip, which is also very useful in fracture mechanics [61]. He also gave the concept of failure mode labeled as mode I, mode II, and mode III, and the essence of this concept is that these three modes can represent all possible crack behavior, and it may be predominantly mode I/mode II/mode III or a grouping of them.

Based on the above-discussed understanding, VCCT criterion was developed by Rybicki and Kanninen [108]. This was proposed as a very good stress intensity factor (SIF) abstraction technique for all three modes because of its good accuracy. Now VCCT is a well-established criterion and effectively calculates delamination. Delamination is the well-known cause of failure, becoming severe or the cause of early failure in the presence of voids. The propagation of cracks/voids initiates the delamination in the laminates, so this failure estimation needs to be considered at application levels [109].

VCCT generally uses linear elastic fracture mechanics (LEFM) based on energy release rate at normal and shear crack-tip deformation mode and compares the energy release rate for interlaminar fracture. Strain energy release rate is the driving force for void (crack) growth to delamination. VCCT assumes that the energy required to separate the surface is equal to the energy required to close the surface. VCCT calculates energy release rate by the function of the crack yielding direction. This method is based on energy release when a crack goes from one position to another [110]. These are the equations to compute the strain energy release rate for modes *I*, *II*, and *III* ($G_{I}$$G_{II}$ and $G_{III}$).

(6) $$\begin{array}{l} G_{I}=-\frac{1}{2\Delta A}\left[F_{y}\Delta v\right]\\ G_{II}=-\frac{1}{2\Delta A}\left[F_{X}\Delta u\right]\\ G_{III}=-\frac{1}{2\Delta A}\left[F_{Z}\Delta w\right] \end{array}$$

**Figure 7:**
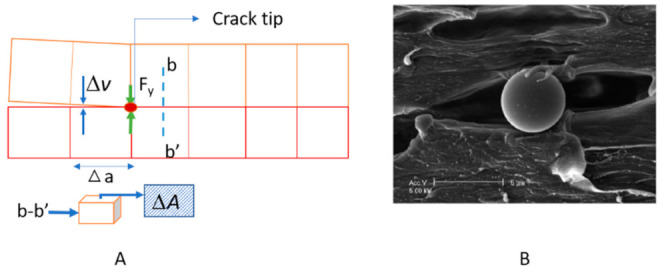
(**A**) VCCT schematic, (**B**) Debond by the voids due to deformation of the pp/glass composite adapted from [111] with permission from Elsevier, 2021.

In the above Equation (6), *F_y_*_,_ *F_x_*_,_ and *F_z_* are the reaction forces in the opening, sliding, and shearing action for modes I, II, and III, respectively. Δu, *v* and *w* is the relative displacement for the action and reaction of mode I, II, and III conditions, respectively, in the local coordinate system and ΔA (in 3D, Δ a in 2D as shown in Figure 7A is the crack extension area. When the energy release rate increases to the critical energy release rate (*F* = *G_I_*/*G_IC_* = 1, when an opening is dominant than sliding and shearing), delamination is started. Figure 7A shows the parameters and schematic of the calculation and shows how void/crack can be taken into VCCT for debonding/delamination, and Figure 7B shows the actual debonding condition by voids because of deformation of the pp/glass composite [111].

As discussed above, voids/crack lead to delamination that’s why it is required to study delamination, and modeling in FEM makes it time and money-saving. Zhuang et al. modeled delamination front void and studied its effect on mode I and II, and it was observed that mode II is profoundly more affected by the voids than mode I [112]. Gliszczynski et al. studied a double cantilever beam test for unidirectional laminate (mode I opening failure) through VCCT and CZM technique with interface element and contact elements [110]. Krueger presented a review on VCCT in different dimensions and elements with equations to calculate energy and stress intensity factors at modes I, II, and III [113]. In the FE model, for analyzing the effect of the voids, it can be placed at a defective location, through that one can observe the void shape and size affecting the energy release rate [114]. This criterion is also a well-known and established technique in research and at the commercial level for predicting delamination behavior [115].

#### 3.2.4. Micromechanics 

**Figure 8:**
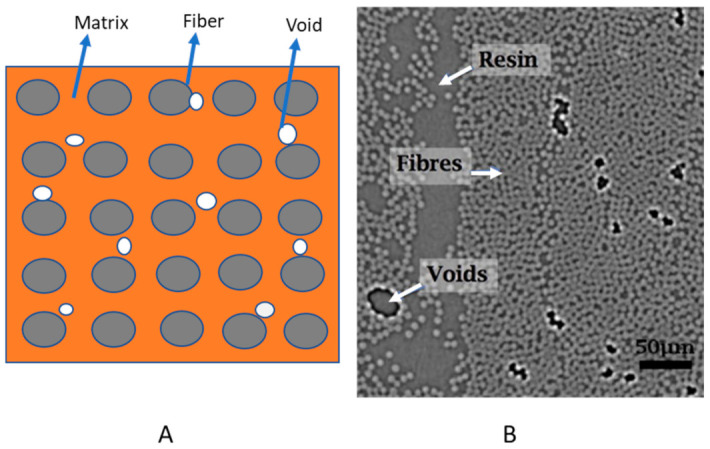
(**A**) Demonstration of RVE model of composite with voids, (**B**) Synchrotron Radiation Computed Tomography (SRCT) image for a micro-scale view of composite adapted from [118] with permission from Elsevier, 2021.

To provide more physical insight into the void’s effect on manufactured components’ failure, micromechanical approaches can be an advantageous because they can cover micro-level manufacturing details. This approach is applied by researchers to obtain the failure behavior. In that direction, Hyde et al. studied both interfiber and matrix voids used in the microstructural model of composites [97]. As per their work and results, cracks start at the inter-fiber voids, and matrix voids strongly influence crack propagation paths. Micro-mechanics or micro-mechanical modeling (representative volume element (RVE)) with defects, using initial crack to fracture failure, can be promising for calculating composite material’s strength at the physical level. For that quantifying the voids with fiber and matrix constituents is one of the important tasks. First, it is necessary to represent the internal structure of composites with manufacturing defects because the voids’ nature is not deterministic. Statistical simulation can be done to quantify defects severity [116]. RVE should be considered based on failure mode and void distributions. To construct the RVE, Ripley’s k function can be used; it defines the pattern recognition of fiber distribution. If the k function is not enough, then the g function from the k function showed the inter-fiber distance to capture the micro-level’s exact irregularity [92,117]. Figure 8A illustrates the RVE (unit cell at micro-scale) covering composites constituents with voids. This is for the demonstration of how RVE could take voids effects, and Figure 8B presents the real micro-scale image (synchrotron radiation computed tomography) to show the voids between fibers and matrix (in the showed figure it is resin) [118].

RVE (unit cell) can be modeled based on scan images or statistical or understanding of fiber, and void fraction in a unit cell, any of the discussed algorithms need well understanding. For FE analysis voids can be modeled as a blank space or elements if the element model it, then assigning low properties are feasible. Elnekhaily and Talreja modeled RVE with defects by using shaking algorithms to make a degree of non-uniformity of fiber distribution depending on their fiber volume fraction [116]. Sudhir and Talreja used a fiber clustering algorithm for considering a defect in RVE construction based on fiber mobility [119]. As micromechanics account for voids (giving a proper space) directly, it affects the stiffness of components at the time of loading. This approach greatly impacts the estimation of failure because it can lead to understanding failure at a very small level and constituents.

One strain and micromechanics-based approach is presented below, named strain invariant failure theory (SIFT), which has good capability to predict failure. The beneficial point of SIFT is micro-mechanical magnification like the micro-strain of laminates amplified by a strain amplification factor which can be predicted by microlevel (RVE) FEM.

Strain invariant failure theory (SIFT):

Strain invariant failure theory (SIFT) proposed by Gosse in 2001 is another precise strain-based failure criterion [75]. Volumetric strain and the equivalent strain are used for failure estimation of fiber and matrix. This technique’s advantage is independence from the coordinate axis, so it does not affect with void’s directions. The theory proposes that when composites are subjected to dilatational (volume) changes or distortional (shape) changes, it creates irreversible deformation. Strain invariants are the basis of this criterion; Tay et al., proposed an element failure concept with SIFT. This concept supports the microlevel study of damage and delamination. Hart Smith also worked on strain-based criteria and SIFT is one of them [79].

SIFT delivers that strain and critical material parameters are related to each deformation mode. These deformation states are related to invariant quantities of the strain tensor, viz. strain and stress correspond in a linear material is ε=s:σ.

**Figure 9:**
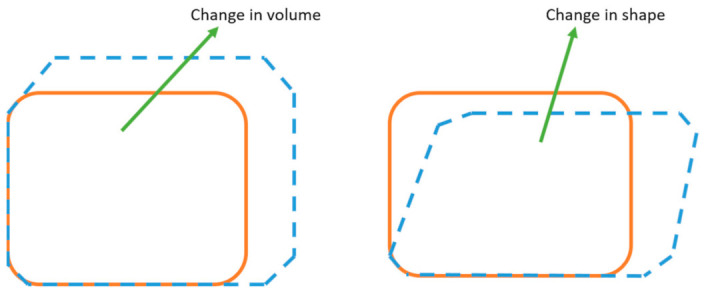
Two variants of SIFT.

The first invariant of the elastic strain tensor and the square root of the second invariant of the strain deviator tensor illustrate the dilatational and distortional strain, respectively. This criterion covers volumetric and distortion deformations, as shown in Figure 9, and Equations (7) and (8) show the invariants ($J_{1}^{\prime }$ and $J_{2}^{\prime }$) for dilation ($\varepsilon_{\mathrm{dil}}$) and distortion($\varepsilon_{dis}$).

(7) $$\varepsilon_{\mathrm{dil}}\left(\varepsilon \right)\equiv J_{1}^{\prime }=\varepsilon_{xx}+\varepsilon_{yy}+\varepsilon_{zz}=\varepsilon_{1}+\varepsilon_{2}+\varepsilon_{3}$$

(8) $$\begin{array}{c} J_{2}^{\prime }=\sqrt{\left\{\frac{1}{6}\left[{\left(\varepsilon_{1}-\varepsilon_{2}\right)}^{2}+{\left(\varepsilon_{2}-\varepsilon_{3}\right)}^{2}+{\left(\varepsilon_{3}-\varepsilon_{1}\right)}^{2}\right]\right\}}\\ \;\;\varepsilon_{dis}\left(\varepsilon \right)=\sqrt{3J_{2}^{\prime }} \end{array}$$

where *Ɛ*_1_,_2_,_3_ is the principal elastic strain tensor, through that effective strains are calculated. Failure occurs if any of the strain invariants (either fiber, matrix, or interphase) exceeds the critical value, as shown in Equation (9). For each strain invariant, critical material allowable is obtained for failure estimation [80].

(9) $$\max\left(\frac{\varepsilon_{\mathrm{dil}}}{\varepsilon_{\mathrm{dil},\mathrm{crit}}},\frac{\varepsilon_{\mathrm{dis}}}{\varepsilon_{\mathrm{dis},\mathrm{crit}}}\right)\geq 1$$

This is also the promising failure criterion at the microlevel, and the strain amplification factor (*M*) is used to connect micro and macro-structure levels. Here, $\varepsilon_{\mathrm{dil},\mathrm{crit}}$ and $\varepsilon_{\mathrm{dis},\mathrm{crit}}$ are the critical dilational and distortional strain.

(10) M=εΔLL0

where *M* is the amplification factor, ΔL is prescribed unit displacement in the analysis, and $L_{0}$ is the micromechanical model’s initial length along the loading direction. Gosse and Christensen proved the reliability of this strain invariants-based failure criteria by collected mechanical test data for different loadings [75]. Due to physical phenomena and intrinsic material properties, SIFT is a promising method in failure theories [120]. Goyal et al. proved the SIFT evaluability for multiaxial lading and showed its FE suitability [121].

The above-suggested criteria are presented to make the predesign strategy and failure estimation in crack (void) conditions. After analyzing the proposed criteria, the below example demonstrates how manufacturing defects (cracks/voids) affect failure. The current example showed the effects of voids in delamination. This presented example shows the effect of voids on the failure mechanism by the suggested VCCT method. 

### 3.3. Example of Composite Laminate with Voids

As it is discussed, that void leads to make early delamination. An example is shown here, which takes the voids into account and estimates the delamination by proposed (VCCT) criterion. This example shows that additional voids affect early delamination between the bonded layers. For that, two cases are studied, first with single voids and second with an additional void near the previous void, to check the effect of voids in the failure mechanism.

**Figure 10:**
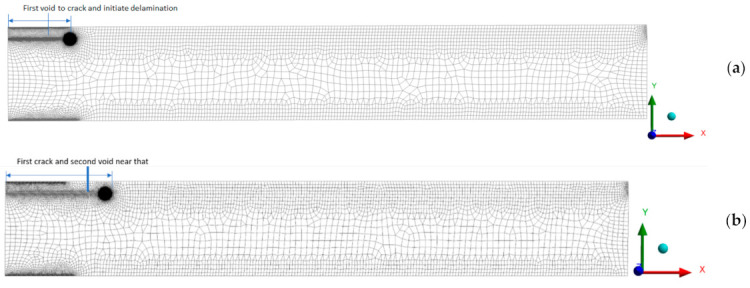
(**a**) Meshed model in case of a single void, (**b**) Meshed model in case of an additional void.

Two layers were modeled as a two-dimensional plate with unidirectional carbon fiber reinforced polymer composite (CFRP). The mechanical properties used for CFRP are E11 = 121 GPa, E22 = E33 = 8.60 GPa, G12 = G13 = 4.70 GPa, G23 = 3.10 GPa, ν12 = ν13 = 0.27, ν23 = 0.4. The plane 182 element was used for meshing the model; as per the VCCT recommendation void region is modeled with pre-meshed crack with a smaller size (the smallest element size is 0.001 mm), as shown in Figure 10. The bottom edge of the plate is fixed, and to analyze the behavior as a double cantilever beam (DCB), 4 kN tension force was applied to the top layer, that’s why, in this case, only the first mode has a significant role. Two models were generated, one with a single void and the second with an additional void near the previous crack, to check the effect of additional void on delamination. The results showed that voids (cracks) easily initiate delamination, and due to additional voids, the first mode energy release rate was highly influenced. The second mode energy rate also increases, but it is less than the first mode, so voids support easy debonding and delamination. In the first case with one void, the critical crack energy release rate (CC ERR) in the first mode is 6 j/m^2^, and due to the additional void, it increases to 16 j/m^2^. Also, CC ERR increase from 0.91 to 4.45 j/m^2^, For mode II. In the first case, a single void created less delamination, which increased in the second case due to an additional void. Hence, voids support easy debonding and delamination.

**Figure 11:**
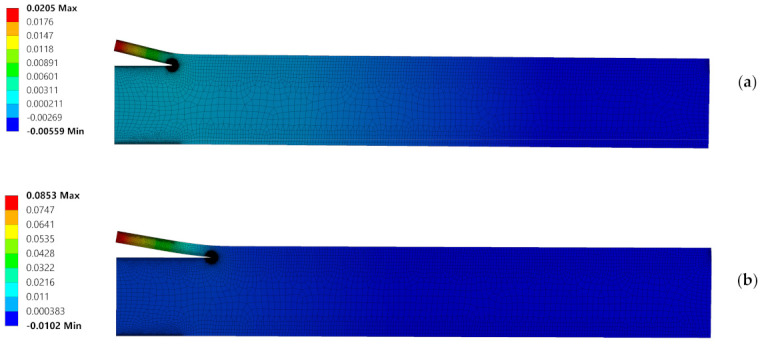
Effect of void in deamination growth (mm) (**a**) Delamination growth in single voids case, (**b**) Delamination growth due to additional voids.

This example was presented to show a voids’ effect on delamination and effect on failure after taking them into account by proposed criterion. It was obtained that additional voids increased delamination length, as shown in Figure 11a,b, and this is also the physical situation that voids lead to delamination, so this criterion took voids effect effectively. This example illustrates the void’s case, which is effective in high voids conditions related to VARTM processed components in a mass production environment, and the suggested criterion (VCCT) was used to consider the effect of the void and check the delamination status in the presence of the new void.

## 4. Discussion

**Figure 12:**
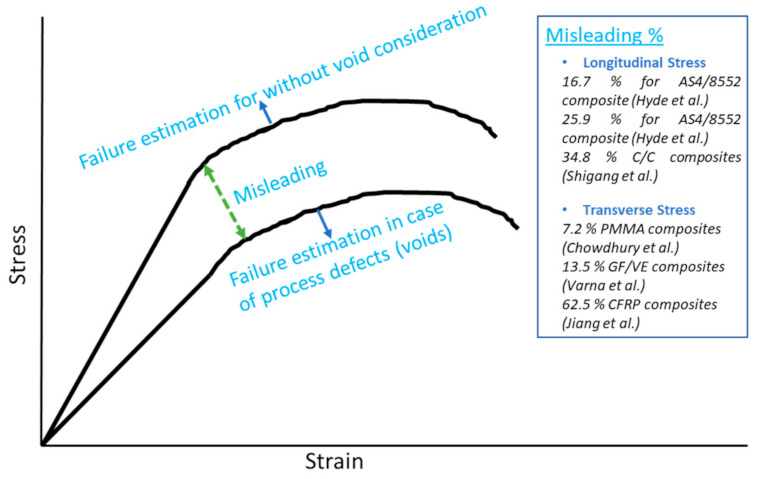
Demonstration of misleading due to Void’s effect.

This paper discussed the failure theories and suggested for VARTM processed polymer matrix composite in mass production environments. The VARTM process is a cost-effective and highly reliable process, but due to void contents, it is necessary to consider void’s (crack) effects in failure theories as a design strategy. It is known that in the case of defects, failure occurred earlier than the calculated strength without void conditions, so voids are important to consider in the design, as illustrated in Figure 12. This figure is presented to demonstrate the misleading condition, and it can be anything in quantity depending on voids, load conditions, and constituents (fiber and matrix) in composites. The misleading percents due to the effect of voids are also included in the figure from the surveyed literature [82,86,89,94,96,97]. This is why, for an effective design, the void’s exact state (from manufacturing conditions) should be considered to save the design from being misleading.

Although some old conventional failure theories are well established, they do not take process defects into account. Recently, researchers have worked on failure theories with process defects. This paper considers void conditions from the manufacturing stage. From the survey, some failure approaches were suggested for failure estimation. These suggested approaches are applicable for primary to advance level design. These well-established selected theories, such as discrete damage mechanics, fracture mechanics (VCCT), and micromechanics, are suggested to design composites. These verified approaches have better capability to capture voids growth and failure. It is suggested to use strain-based theory like TMSC for primarily level design to get the first estimation because it is in a simple form and the extension of well-known maximum principal strain criteria, after passing from it, can be checked by any of advance level discussed theories. In these cases, VCCT is suggested for delamination due to voids. Discrete damage mechanics is concluded for the advanced level as damage mechanics because its calculation is based on the crack (void) density and its function. Further, it is independent of mesh details.

Micromechanics is also an excellent approach to get micro to macro-level failure, so it is presented that RVE (unit cell) can model with void consideration. This approach is discussed because it takes the small level manufacturing details and analyses the failure at the micro-level. This estimation supports the precaution strategy and preunderstanding for macro-level safety. Finally, one example is presented for delamination because it is the most known cause of failure in composite laminates and estimated the effects of voids on delamination by proposed criterion. One more criterion is suggested, named SIFT, which accounts for strain and micromechanics to cover details with accuracy. Overall, it can be obtained that this paper gives a better idea to choose the failure theory for composites. Researchers and industrialists can choose the theory according to the manufacturing and practical conditions.

The failure theories with manufacturing defects enhance the reliability of the composite design. It so happens that a cost-effective VARTM process is possible for mass production with enhanced failure understanding due to process conditions. If voids have undesirable effects, then the process should be revised according to the void’s conditions. After estimating the strength with voids, it can be understood that components are useful or not. It is good to use a reliable failure theory for designing. It can be valuable for each composites’ application. Design with manufacturing defects needs a less costly process, so FEM is the best tool for predesign to check approximate conditions of manufactured components under application loading, and suggested theories are well-recognized in FEM. This review gives a better vision into choosing failure theories for composite materials with the manufacturing process and practical conditions, i.e., defects (voids). After understanding the selected known technique, new criteria can be proposed to obtain all the conditions’ premises at the application level with process defects.

## 5. Conclusions

Although the composite manufacturing field has reached an advanced stage, optimizations are still underway as it is implausible to manufacture defect-free composites. So, defects should be considered in failure theories to estimate the design conditions accurately. VARTM is a good technique for polymer composite fabrication with a high production rate and good quality. However, it suffers from voids defects in fabrication. Also, high cost and sophisticated accessories techniques cannot manufacture completely void-free composites. Therefore, it is necessary to consider voids defects in the failure approach for a durable design. This review presented the previous work on failure theories that do not consider the effect of voids. Then, due to the essence of void consideration, we reviewed the researches that considered void effects in failure. It was obtained from a survey that strength is diminished by the increment of void content, as 1% and 2% percent increases in voids, accompanied by decreases of 2.5% and 12.7% in tensile and flexural strength, respectively [41,90]. The strength reduction in different directions depends on loading conditions. Figure 6B shows the void effect on strength in different composites and directions. Finally, based on surveyed understanding, theories were suggested to consider the effect of voids on strength. This covered damage mechanics, fracture mechanics, and micromechanics. Moreover, this is well-established with the finite element technique that is time-saving, less costly, and good for design strategy. An example was presented with the suggested failure theory to show the effect of voids on strength and the suggested theory can assess the void effect numerically. Due to voids, delamination length was increased, and the energy release rate increased significantly (6 to 16 j/m^2^) in mode I with additional voids. Table 3 summarizes the suggested methods and reasons to suggest.

It is concluded from this review that for the reliable and durable design of VARTM processed composites, a consideration of process defects (voids) is necessary. This paper suggests failure theories in void states and gives a future idea for the better design and prediction of strength at a quality level. A few meaningful points are highlighted for an accurate design of cost-effective VARTM processed composites:

1. Voids are significant defects in the composite manufacturing process (VARTM). Readers will understand the selection of failure theories in a cost-effective environment (mass-production) at the application level (working environment because different loading makes a different kind of failure) with the presence of voids.
2. Voids to delamination should be considered at the time of design for composite laminates. Fracture mechanics-based VCCT failure estimation was proposed for the delamination study because it can consider the voids’ effect, enabling the energy release rate.
3. Preliminary and damage level failure prediction is essential. Checking for preliminary level design viz. preliminary failure theories (TMSC) without considering damage behavior is enough then consideration of damage and crack behavior by DDM will be a good practice for precise understanding because DDM can consider voids by their density.
4. Micromechanics with failure theory is also an excellent option to model defects (voids) for failure prediction at the micro-level and supporting a relation from micro to macro-level.

## Figures and Tables

**Table 1:** Steps for process completion of VARTM with voids’ reason at a certain step.

Process	**Pre-Infusion**	**Infusion**	**Post-Infusion**
	• Mold preparation • Material placement (fiber reinforcement) • Maintain flow distribution medium, injection port, vent port, vacuum bag against the sealing tap • Debulking process (optional)	• apply the vacuum inside the bagged preform assembly • Fill the resin into the resin reservoir. Keep the vacuum port on	• Close the injection port • After curing resin, turn off the vacuum then demold the composite part
Defects (Voids) reason	• Moisture in resin • Resin mixing and degassing and bag integrity • Leak rate	• Resin flow, pressure, flow timing, and thickness management	• Injection and vacuum off timing, • demolding conditions

**Table 2:** Some well-known conventional failure theories and their approaches.

Author, Year	Research Approach/ Failure Theory	Advantage	Disadvantage	Remark
St. Venant, 1855 [56,57]	Maximum strain failure theory	Simple to use because of direct comparison with ultimate strain	Non-interactive and developed for isotropic cases	Strain based criteria
Rankine, 1857 [58,59]	Maximum stress failure theory	Simple to use because of direct comparison with ultimate stress	Non-interactive and developed for isotropic cases	Stress based criteria
Griffith, 1920 [60]	Linear elastic fracture mechanics	First fracture theory based on energy release rate (g criteria belongs)	Developed for isotropic material and limited for a single crack, no nucleation accounted	Brittle fracture (strength depends on the size of cracks)
Norris, 1946 [43]	Second power interaction formula for ultimate strength	Consider orthotropic material	Very basic formulation as von-mises and Bauschinger effect was not accounted	Plywood (tension-compression and shear test)
RHill,1948 [44]	Polynomial criteria extension of von mises	Included more term for accuracy and generalization of a Hubris-Mises criterion	No. of unknowns were more, and no Bauschinger effect considered	Yielding and plastic flow of an anisotropic metal.
Irwin fracture theory, 1957 [61]	Plastic zone consideration in fracture mechanics	Extended Griffith theory, and related to the concept of crack intensity factor	Firstly extended for quasi-brittle materials and no nucleation of a crack	Extend Griffith to ductile for plastic zone consideration
Dugdale’s Model, 1960 [62]	Elastic-perfectly plastic Tresca yield criteria	For damage cohesive energy-based model capable of nucleation of the crack	Parameters are dependent on hit and trail, based on remeshing and predefined area of the crack	Cohesive zone model
Tsai, 1965 [63]	Quadratic function of stress	Interactive theory covered laminated effect	No Bauschinger effect accounted	Extension of distortion theory for anisotropic material
Hoffman, 1967 [64]	Fracture condition	Adopted Phenomenological fracture conditions	validity range is limited for material groups, i.e., brittles	Hill’s yield condition(extension)
Tsai-wu, 1971 [45]	Polynomial (interactive)	Interactive and most valid among polynomial criteria	The interaction term needs experimentations	Scale function of two strength tensor
Whitney-Nuismer failure criterion, 1974 [65]	Tensile strength and characteristic dimension (different then linear elastic fracture mechanics (LEFM))	Failure prediction along with the thickness of the laminate	Limited to certain features and geometries	Stress based criteria
Hashin, 1980 [46]	Quadratic stress polynomial	Considered different failure modes of fiber and matrix	Certified for unidirectional and specific loadings only	Failure mode
P.W.Mast 1995 (I,II,III), [66,67]	Energy density approach	Damage estimation by the dissipation of energy so internal failure can capture	Specified for submarine structures	Strain-induced damage
Puck, 1969 and 1996 [49]	Fiber and inter-fiber failure mode	Various failure mode can be obtained	Discontinuities for different load combinations and failure modes are non-fatal	Physically-based failure criteria
Edge, 1996 [68]	Grant sanders method	Ply by ply failure estimation, mode and location can cover, discrete failure phenomena	Discriminations in high strain condition and matrix-subjected configurations	Stress based criteria
Christensen theory, 1997 [69]	Matrix (mode I) and fiber (mode II) dominated failure	Two modes for fiber ad matrix dominated composites with micromechanics hints	Particularly specified for polymer composites	Stress based criteria yield
Cuntze, 1997 [70]	Inter fiber failure mode and internal friction values	Covered a large variety of fracture and failure with micro and macro-level	The modeling of some invariants are tricky, and probabilistic terms were used	Invariant failure mode concept
Eckold, 1998 [71]	Pragmatic approach (Design environment)	Failure definition in design condition with initial failure	The approach was application-specific, and design was based on assumptions	Based on application problem to tackle design
Butalia, 1996,2001,2012 [72,73,74]	Maximum strain progressive laminate failure ply discount method	New strain energy-based failure method with lamination consideration	Specified for particular loading conditions	Strain energy-based criteria
Gosse, 2001 [75]	SIFT (Strain invariant failure criteria)	Covered wide range with the effect of volumetric and equivalent strain, included micro-level consideration	Developed for polymer composites and assumed that both failure modes (fibers and matrix) are independent	Strain based criteria
Yeh,2002 [76]	Quadric surfaces criterion (biaxial test has reduced)	Extension of Tsai-wu polynomial criteria and no bi-axial test needed	Specified for certain loading cases	Strain based criteria
McCartney, 2003 [77]	Assessment of failure criteria	Proposed crack prediction model	Applicable for specific cases and reliable only in mode I cracking	Physically-based damage model
Bogetti, 2004 [78]	Progressive laminate failure (maximum principal strain and ply discount)	Progressive failure in laminate with non-linear considerations	Specific for particular loading conditions	Strain based criteria
Tay, 2005 [79]	Element failure method (damage progression)	SIFT theory extended with element failure concept for damage progression, delamination, and considered micro-level details	Verified for the certain load cases	Strain based criteria
Hart-Smith, 2010 [80]	SIFT (for metal and polymer composite)	SIFT extended for metals	There are already well-defined criteria for metals, no specific need	Strain based criteria

**Table 3:** Suggested failure theories.

Suggested Failure Theories	Reason of Suggestion
Discrete damage mechanics [103]	Advance level design to consider crack/void density for analysis, and the main advantage is mesh independent.
Fracture mechanics (VCCT) [113]	To estimate voids growth to delamination (delamination approach). The pre-meshing definition can model voids.
Micromechanics [97,119]	To capture micro-level to macro-level failure with modeled voids which can capture microvoids as well.
TMSC and SIFT [75,99]	For a primarily level design checking strain-based TMSC approach is suggested because stress is effected due to change in young modulus in case of voids. SIFT is a strain-based and micromechanics included criterion, which add details from micro to macro-level.
